# Implementation and Evaluation of Vision-Based Sensor Image Compression for Close-Range Photogrammetry and Structural Health Monitoring

**DOI:** 10.3390/s20236844

**Published:** 2020-11-30

**Authors:** Luna Ngeljaratan, Mohamed A. Moustafa

**Affiliations:** Department of Civil & Environmental Engineering, University of Nevada, Reno, NV 89557, USA; lngeljaratan@nevada.unr.edu

**Keywords:** image compression, non-adaptive interpolation, wavelet transform, photogrammetry, accuracy, vision-based sensor, optical targets, structural health monitoring

## Abstract

Much research is still underway to achieve long-term and real-time monitoring using data from vision-based sensors. A major challenge is handling and processing enormous amount of data and images for either image storage, data transfer, or image analysis. To help address this challenge, this study explores and proposes image compression techniques using non-adaptive linear interpolation and wavelet transform algorithms. The effect and implication of image compression are investigated in the close-range photogrammetry as well as in realistic structural health monitoring applications. For this purpose, images and results from three different laboratory experiments and three different structures are utilized. The first experiment uses optical targets attached to a sliding bar that is displaced by a standard one-inch steel block. The effect of image compression in the photogrammetry is discussed and the monitoring accuracy is assessed by comparing the one-inch value with the measurement from the optical targets. The second application is a continuous static test of a small-scale rigid structure, and the last application is from a seismic shake table test of a full-scale 3-story building tested at E-Defense in Japan. These tests aimed at assessing the static and dynamic response measurement accuracy of vision-based sensors when images are highly compressed. The results show successful and promising application of image compression for photogrammetry and structural health monitoring. The study also identifies best methods and algorithms where effective compression ratios up to 20 times, with respect to original data size, can be applied and still maintain displacement measurement accuracy.

## 1. Introduction

The advancements of non-contact and vision-based sensors in the field of structural health monitoring (SHM) as well as the development of optics and computer vision algorithms have led to a growing demand, among the civil and construction engineering communities, for long-term continuous and real-time vision-based SHM. Currently, monitoring using vision-based sensors incorporates an offline camera calibration and a closed-range photogrammetry process while using either artificial markers [1,2,3,4,5,6,7] or relying on the natural features of the structure [8,9,10]. Several previous works have shown the robust development and promising future of vision-based sensors deployment for SHM purposes [9,11,12,13,14,15]. Meanwhile, the development of the sensor system for long-term continuous and real time monitoring is still underway. Vision-based sensors capture two-dimensional data, i.e., raw images, dictating the size of the storage that leads to a large concern of excessively large storage when long-term or continuous monitoring is desired. Using high-resolution cameras is also mandatory in some applications that require a high accuracy in distance or displacement measurements but does not emerge as a cost-effective solution. Another concern is associated with the pressing need for fast data transmission and efficient algorithms for image processing and analysis when vision-based sensors are to be deployed in the future for real-time monitoring. 

The goal of this study is to contribute towards enabling future real-time or near real-time SHM using vision-based sensors through robust digital image data compression. The objective of this study is to reduce data storage of vision-based sensors and investigate the viability and feasibility of using compressed digital image data in photogrammetry, displacement measurements, and vibration-based SHM. The accuracy of vision-based sensor metrology highly depends on the image resolution and scale [16]. Therefore, it is essential to assess and quantify the effect and implications of the applied compression on digital images when output images are used for photogrammetry and SHM purpose. Specific definitions of what is meant by photogrammetry and SHM in relation to vision-based sensors are provided in the next section. The applied compression method essentially optimizes the data storage by minimizing the number of required pixels that is used to represent an image. For example, a monochrome image with a dimension of 2560 × 2048 pixels contains n = 8 bits depth so it stores $2^{n}=256$ possible black (0)-to-white (255) color information of the image that results in 5.2 MB size for a single image. As the SHM duration increases such as 1-h of bridge monitoring under traffic loading with 30 frames per second (fps) rate, the required data storage increases to 561.6 GB since 108,000 images have to be stored based on the selected fps.

The idea of data compression and data loss recovery has been already implemented for time-domain-signals, or time signal for short, of wireless sensors network using the compressive sensing (CS) algorithm [17,18,19,20,21,22] for instance, which can be integrated in the monitoring system [21,23]. Wavelet-based transforms are also widely used tools in time-signal compression from vibration-based SHM data for the purpose of civil structure condition monitoring [24] or the identification of structural modal properties [25]. However, for image compression, the applications are available mostly in the field of satellite imagery [26,27,28,29,30,31], medical imaging [32,33,34,35], or in aerial photogrammetry [36,37,38,39,40,41]. Each field also proposes a compression method that is considered suitable for each area of application. Hyperspectral image compression for example, is the essential concept in the remote sensing field not only to compress the bulk data, but also to store them in details [42,43,44,45,46,47]. A new standard, JPEG 2000, has also been developed to provide the framework of still image compression with wide-range of applications including remote sensing and medical imagery areas [48,49,50,51,52,53,54,55]. Combinations between the hyperspectral image compression with principal component analysis (PCA) and JPEG 2000 perform faster compression that are benefit for real-time applications [56,57,58,59,60,61,62,63,64]. Meanwhile, very limited studies have been reported relate to image compression implementation in the field of vibration-based SHM. 

The existing literatures in SHM that implement image compression methods are mainly for structural health diagnosis or for training purposes using advanced convolutional neural networks (CNNs) or deep learning frameworks. Comprehensive reviews of image and video compression with neural network can be found in Dony et al. [65], Jiang et al. [66] and Ma et al. [67]. Studies conducted by Yang and Nagarajaiah [68] and Huang et al. [69], reconstructed SHM images based on the CS theory that crack information in a structure is more pronounced and exhibits sparsity in the image. Xu et al. [70] resized the resolution of rectangular raw images of damaged reinforced concrete columns into smaller square pixels image to reduce calculation costs on the supervised learning procedure. Su [71] downsized 100 sampling images that were used in training model for concrete pavement to reduce computational time. Xu et al. [72] resized the height pixel unit of grayscale images using bicubic interpolation then cropped them into smaller elements as the input of the deep network. These data were used to train the machine algorithms for identification framework of steel surface cracks. 

In vibration-based SHM, the latest study by Ri et al. [73] used downsized image data from a bridge-field experiment. The study demonstrated that JPEG image quality of 20% was the threshold to balance between an effective compression and the measurement accuracy. However, applying image compression using the CS technique to the digital images registered using a sub-pixel algorithm such as in the digital image correlation (DIC) method was likely to be inefficient [74]. It was found that processing thousands of camera images was prohibitively expensive, and should be preceded by another technique, such as a shape description method for potential application in in-situ data compression. A study by Akcay [75] also stated that compressing images that used sub-pixel registration resulted in significant errors, especially in computing exterior orientation and generating 3D point clouds since sub-pixel registration required high resolution of images. To summarize, digital image compression from vision-based sensor for vibration-based SHM purposes remains an open field for further studies and implementations, especially for vision-based SHM and using the principal of sub-pixel registration in measuring the displacement. Thus, there is a need to explore the feasibility and novel methods of applications of various advanced, yet computationally efficient, image compression algorithms. A comprehensive assessment of such methods is needed when applied to compress large image data files in high compression ratios. There is also a need to study the effect of data compression on the digital image photogrammetry and measurement accuracy for SHM purposes. These aforementioned knowledge gaps is what this study attempts to fill. 

In this study, an overview of the vision-based sensor system using optical points or targets as well as the sensor deployment in photogrammetry and SHM process is explained first in Section 2. Section 3 provides the proposed image compression framework and characterizes the selected image compression method with the assessment of the image quality using several statistical indices. Section 4 describes the systematic implementation of the proposed image compression framework, from the compression to accuracy assessment, using a one-inch steel block experiment. Section 5 focuses on the static and dynamic displacement measurement accuracy assessment of the proposed image compression frameworks from the monitoring of a continuous quasi-static experiment and a unique full-scale seismic shake table experiment. Finally, Section 6 provides the key takeaways and conclusions of this study. 

## 2. Vision-Based Sensor System for Photogrammetry and Structural Health Monitoring

### 2.1. System Requirements

**Figure 1:**
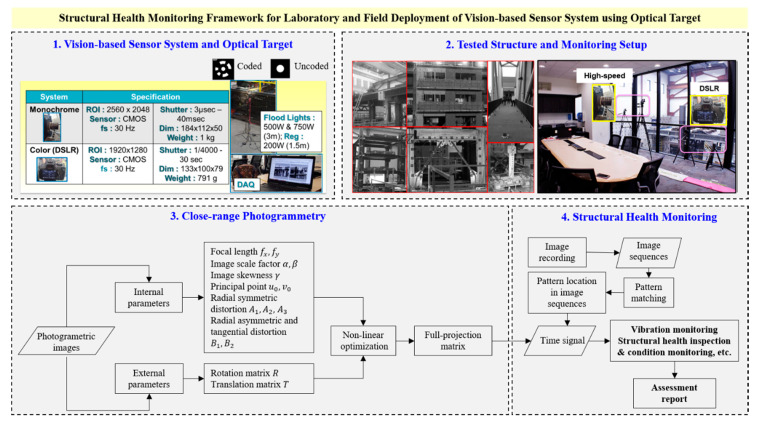
SHM framework and various components of vision-based sensor system for laboratory and field deployment using optical targets.

The vision-based sensor deployed in this study uses two sets of monochrome cameras: consumer grade digital cameras and exclusive high-speed cameras. Each set comprises two cameras to allow three-dimensional (3D) monitoring and measurements. Complementary metal oxide semiconductor (CMOS) image sensors are commonly used in the camera system with image resolution ranging from 5 to 18 Megapixels or more, depending on the camera specifications. The photogrammetry images are stored as .tiff or .jpg formats, and test images are sometimes converted from video format when commercial digital cameras are used. For indoor monitoring, lightings are sometimes required but they are not necessary for outdoor or field monitoring. The setting exposure and sampling rates are varied depend on the environmental condition and monitoring types. For vibration SHM purposes they are normally set to f/16, and used fast shutter speed to avoid motion blur. However, lower sampling rates such as 30 fps are sufficient for vibration monitoring or for structural system identification of the monitored structures [4,5]. Several types of optical templates are available but this system uses a template of a white dot surrounded by white rings printed on a black background based on Schneider [76] design as shown in Figure 1. The geometry of the white rings are varied for the ease of identification as each geometry translates into a different number and it is identified as a coded target. The single white dot does not specify a certain number, but also has a similar function as the coded ones. The behavior of the structure is detected through the movement of center of the optical targets, sometimes referred to as templates, points, or dots. These targets are rigidly attached and randomly distributed to the structure within the field of view (FOV) and area of interest as dictated by the monitoring setup. For large-scale experiment, the monitoring requires a distant setup to accommodate the large FOV, so the system completely depends on the environment where the experiment is being conducted. The different components of the full vision-based system along with the various processing steps are presented in Figure 1. As shown in the Figure, large-scale monitoring setups might need to be located at higher platform to monitor a broader area and to get a clean view of larger structures. 

### 2.2. Photogrammetry Procedures

The SHM using the proposed system starts by the photogrammetry process, in which the internal and external camera parameters need to be determined. In close-range photogrammetry, the procedure of finding the internal and external camera parameters generally adopts the method established and used in many studies, such as Tsai [77], Heikkila and Silven [78], Ruther [79], Fraser [80], Gruen [81], and Bouguet [82], to name but a few. This procedure is usually referred to as close-range photogrammetry or photogrammetry calibration. In most SHM applications using vision sensors, camera calibration is specifically dedicated to remove lens distortion [83,84]. The camera calibration in this study is in fact a photogrammetry deliverable with the final goal of defining the relationship between the two cameras by forming a full-projection matrix. In the rest of the paper, just the term photogrammetry is used for simplicity to refer to the process described herein. 

**Figure 2:**
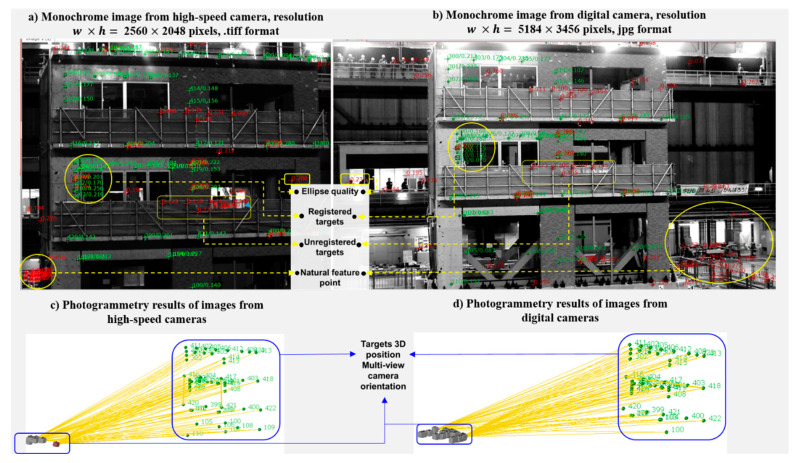
Example of photogrammetry images with typical output; monochrome images captured from a three-story building seismic test monitored using high-speed and digital cameras, and respective photogrammetry results for 3D positions and rays of optical targets in object coordinates.

The internal and external camera parameters defined in the photogrammetry process are listed in Figure 1. These parameters are used to compensate for the systematic error in the camera sensor, either CCD or CMOS sensor. They are recovered from a set of photogrammetry images using self-calibrating bundle adjustment [85]. Bundle adjustment starts with the least square estimates of the internal orientations. Then, by using these estimates, all parameters are determined simultaneously using the iterative non-linear collinearity optimization to minimize the error in each image plane. The additional parameters, i.e., radial symmetric, asymmetric, and tangential distortions are also computed in the bundle adjustment process. The overall process requires only a set of static images, that could be as little as five and up to as many as hundred images, taken from multi-view and different angles toward the structure as shown in Figure 2. Other statistical qualities of the images such as the ellipse quality, image deviation, or targets position in the 2D and 3D coordinates as shown in Figure 2 can also be quantified from the photogrammetry process. The figure shows examples of the additional output of photogrammetry: (1) registered targets, i.e., targets that are selected to track the structure movement; (2) unregistered targets, i.e., targets that are used in photogrammetry but neglected in SHM as their locations are not within the scope of monitoring but their attachments are required for bundle adjustment convergence; (3) ellipse quality is the grayscale quality of the identified targets; (4) natural feature point is the point that is found using the ellipse finding algorithm but their locations are also not within the scope of monitoring;

The photogrammetry procedure is then followed by the SHM analysis, as illustrated in Figure 1, in which videos or continuous images are recorded and stored in the system. They are post-processed further using the sub-pixel registration of pattern or template matching method [86] that is used to track the target locations within image sequences. When the SHM is conducted using video recording, a conversion to image sequences is preferable so the results from the imaging photogrammetry can be used. During conversion, the SHM image resolution sometimes has to be adjusted to be similar to the resolution of the photogrammetry images, or vice versa. Finally, using the relationship between two cameras (as full-projection matrix) and the change of target location in each image (from the pattern-matching method) as outlined in Figure 1, images are translated into time-domain response signals, i.e., displacement, velocity, or acceleration. These signals are certainly the SHM data which are then used to assess and report the condition of the structure, identify structural modal properties, etc. depending on the purpose of the SHM and user needs.

## 3. Proposed Framework for Digital Image Compression

### 3.1. Overview

This study is not aimed at developing new image compression methods or algorithms, but rather to assess and extend existing methods when specifically tailored to the application in hand. The next goal is then to systematically explore the effect of image resampling on the photogrammetry and apply it for SHM purposes. In other words, we utilize the most applied yet computationally simple resampling algorithms. By tuning the influence of each of the different algorithm to the compressed image quality, the photogrammetry results and SHM accuracy can be easily tied to assess the effectiveness of the selected algorithms.

The term “compression” in this study is defined as reducing the size of the digital image, in terms of storage size, by a scale factor, i.e., the ratio between the size of the original data and compressed data. In the digital image up or down scaling, two categories of digital image processing are generally used, i.e., resizing and resampling techniques. Image resizing changes the image size while keeping the pixel dimension constant while image resampling physically changes the number of pixels in the image. Since the compressed images are further used in the photogrammetry and vibration-SHM for the vision-based sensor, the image resampling technique is a possible option as it reduces the data storage without losing the image original quality such as creating blurry or distorted images. The non-adaptive image interpolation is a resampling technique that is mostly preferable for real-time applications because some other adaptive image interpolation algorithms require longer processing time [87]. They also consume more hardware resources as compared to the non-adaptive image interpolation algorithm. Several interpolation algorithms have been used for image resampling, from the simplest nearest neighborhood to more complex functions such as cubic splines [88]. In the frequency domain, wavelet transforms also offer advantages in compressing and reconstructing images without high hardware cost [89] as they are easier to implement than the Fourier method [90] and provide easier computation and transform processes [91]. The compression rate or the ratio between the original data volume from the input image with full resolution and the compressed data volume obtained after resampling process is expressed by Equation (1):

(1) $$I_{CR}=\frac{original\;data\;volume}{compressed\;data\;volume}$$

**Figure 3:**
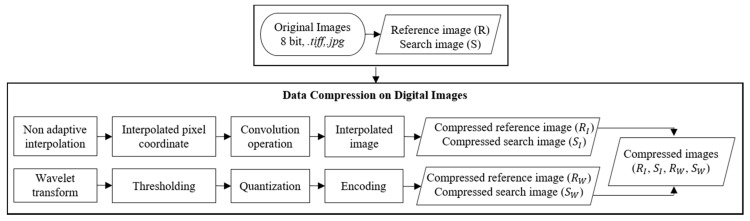
Proposed framework for digital image data compression.

The $I_{CR}$ in this study is expressed from one single image ratio before and after compression and it is also associated to the total volume of images recorded from SHM and stored in the system. The proposed framework for digital image data compression is shown in Figure 3. The procedure is outlined for monochrome image with n= 8 but can also be extended for the application on colored images with higher bits, such as 24 bits or 32 bits. The process starts by resampling the photogrammetry images either in .tiff or .jpg format as taken by the two cameras in the system. For the rest of the paper, the frames taken by the first camera are referred to as reference frame or reference image, R, and the ones captured by the second camera as search frame or search image, S. These are associated with the terms used in the sub-pixel pattern matching method to track the target location between the two frames when processing the SHM images. The term input images refer to the initial image with full resolution of 2560 × 2048 pixels while compressed images refer to the output image of the applied resampling algorithm. 

How the resampling algorithm works in compressing an image is briefly described as follows. Considering a pixel i(x,y) where x,y are the horizontal and vertical coordinates of pixel i located on an image I with a size of X×Y pixels. Image I is resampled using scaling factors $r_{x}$ and $r_{y}$ in x and y directions so $r_{x}=r_{y}$ when the scaling factor in both directions is similar. After resampling, the image is repositioned by rotating the image by angle θ and the new coordinate of pixel i(x,y) is now $j\left(x^{\prime },y^{\prime }\right)$. The latter is calculated by adding a translation vector $t_{x}$ and $t_{y}$ so the pixel i(x,y) can shift to the new location $j\left(x^{\prime },y^{\prime }\right)$ along a straight-line path. The overall process is expressed as the geometric transformation expressed in Equation (2):

(2) $$\left[\begin{array}{c} x^{\prime }\\ y^{\prime } \end{array}\right]=\left[\begin{array}{c} x\\ y \end{array}\right]\left[\begin{array}{cc} cos\theta & -sin\theta \\ sin\theta & cos\theta \end{array}\right]\left[\begin{array}{cc} r_{x} & 0\\ 0 & r_{y} \end{array}\right]+\left[\begin{array}{c} t_{x}\\ t_{y} \end{array}\right]$$

According to Shannon’s first theorem, the lower and upper bounds of $I_{CR}$ is expressed as $\frac{n}{H\left(I\right)+1}<I_{CR}<\frac{n}{H\left(I\right)}$ in which n refers to the bit depth and equals 8 for monochrome image and H(I) is the image entropy that is explained later in Section 3.4 when assessing the quality of compressed image. Even though high compression ratio can be possibly achieved based on $I_{CR_{max}}$, digital images are restricted, in either compressing or magnifying, without compromising the degradation of their pixel values. Therefore, besides the criteria of fast algorithm and high compression ratio, maintaining the image quality is significant, especially for photogrammetry, SHM, and accurate displacement measurement as desired in this study. Two resampling methods using interpolation and wavelet transform are selected with detailed description described in the next section.

### 3.2. Non-Adaptive Linear Interpolation

Interpolation algorithms are basic resampling techniques that reduce the input image size by estimating the new pixel intensity of the compressed image based on the neighboring pixel values. In this study, interpolation is selected as the compressed images can be presented without any visual loss [92] and they are divided into two types: adaptive and non-adaptive techniques. The non-adaptive image interpolation is utilized here since it is mostly preferable for real time applications. Image interpolation is applied in two directions, x,y, and attempts to reach the best approximation of a pixel’s intensity based on the values at surrounding pixels [87]. 

The non-adaptive interpolation scheme is operated using interpolation Kernel $K_{1}\left(t\right)$ with properties such as regularity, moments, and symmetry. For monochrome images, they use adjacent pixels from 0 to 255 or more when interpolating. Four functions of non-adaptive linear interpolation, i.e., bicubic, bilinear, box kernel, and Lancsoz are selected in this study. The simplest method is box Kernel that takes a region of bandwidth h that controls the degree of smoothing. It is specified as 0.5 in this study and the interpolated pixel value is computed by taking the pixel average in the specified region. The algorithms become more complex and require longer computational time when they use more adjacent pixels, but the results are more accurate. Bilinear interpolation is more complex than box kernel as it considers the known 2×2 neighboring pixel values. Bicubic considers the closest 4×4 pixel values so the interpolated image is smoother and generates sharper images than box kernel or bilinear method. Another interpolation method uses Sinc kernel with very high accuracy in smooth data. However, it produces ripple around the image edges since the function sinc(t) decays at a rate of 1/t that creates the Gibbs phenomenon or ripple throughout the image [93]. The solution is to apply a window function to limit the ripple artifact and the most commonly used method in image processing is a Lanczos window with a positive integer n= 2 or 3. These algorithms are applied on the input images to calculate the interpolated pixel coordinate then the convolution operation is performed to obtain the pixel intensity value of the compressed images as outlined above in Figure 3. 

### 3.3. Wavelet Compression

Wavelet transform is a widely used method in the image and video compression applications. The general architecture of the wavelet compression is outlined in Figure 3 where a wavelet transform or encoder is selected first and then operated by transforming data to remove redundancy in the image. The transform coefficients are quantized followed by the entropy coding of the quantizer output. The wavelet compression algorithms used in this study are in the category of progressive coefficients significant method (PCSM) and coefficients thresholding method (CTM). PCSMs includes the adaptively scanned wavelet difference reduction (ASWDR), embedded zero tree wavelet (EZW), set partitioning in hierarchical trees (SPIHT), spatial-orientation tree (STW), and wavelet difference reduction (WDR) algorithms, while CTM uses subband thresholding and Huffman encoding or LvlMmc. 

EZW was first introduced by Shapiro [94] and became the first most powerful wavelet encoder for image compression. It forms a tree structure and uses a list of significant coefficients. After applying a discrete wavelet transform (DWT) on the image, the roots are located into the lowest frequency sub-band of the wavelet tree. A threshold value is predefined to be compared with the wavelet coefficients. The SPIHT [95] encoder provides the same basic progressive concept as EZW but it uses three lists of significant pixels, insignificant pixels and insignificant sets. While EZW is the first encoder that provides a significant rate-distortion performance, the SPIHT encoder is also a fast algorithm that improves the EZW performance by proposing new encoding for the position of the significant coefficients [96]. STW is essentially the SPIHT algorithm but it uses a state transition model in organizing the output of its coding. ASWDR [97] is relatively recent algorithm and is also the adaptation of WDR [98] that adds one last step of creating new scanning orders. While WDR uses a fixed ordering, ASWDR employs a varying order so it can adapt with a specific image feature creating objective computation and improving the compressed image quality. 

A note related to the wavelet compression applied in this study is that the pixels of the input image must be the powers of two. Since the width of the input image used in this study is 2560 pixels, which is not the power of 2, it requires scaling in the horizontal direction before applying the wavelet compression. For this purpose, the bilinear interpolation is selected due to its simplicity then the wavelet compression is applied after horizontal scaling following the procedure in Figure 3.

### 3.4. Compressed Image Quality Index

A histogram of a compressed image quantifies the information contained in the output of image compression from the resampling algorithms described above. The Shannon entropy of image I, H(I), is calculated using Equation (3) with p(i) as the probability mass function that contains the normalized histogram gray level counts of the compressed image. It is always non-negative and indicates the number of bits on average required to describe the random variable. Higher entropy indicates the variable contains more information and in turn, the compressed image will be more detailed. Entropy reaches the maximum value when the probability distribution is uniform. Note that there is a relationship established between image entropy and compression ratio as briefly mentioned before. The lower and upper bound of compression ratio based on Shannon estimation, i.e., $\frac{n}{H\left(I\right)+1}<I_{CR}<\frac{n}{H\left(I\right)}$, clearly shows the relationship between entropy H(I) and $I_{CR}.$ The higher the entropy, the lower the compression ratio that can be achieved, and vice versa. That relationship is not explored further in this study as the applied compression ratio in this study is much higher than the theorem:

(3) $$H\left(I\right)=-\underset{i\in I}{\sum }p\left(i\right){log}_{2}p\left(i\right)$$

A number of indexes have been proposed in the literature to measure the image quality degradation analyzed by resampling or compression algorithms. The simplest metric implemented in measuring the image quality is the peak signal to noise ratio PSNR that is given by Equation (4). The variable n is the bit depth of the image and for a monochrome image; it is defined as 8-bit. MSE is the mean square error between the compressed and input image; PSNR is 48 dB without MSE or higher when MSE is included. Both PSNR and MSE are computed based on pixel-to-pixel difference so they provide simple computation with clear physical meaning. Another metric selected in this study is the structural similarity index metric SSIM, which is computed using Equation (5). It measures the similarity between two images in terms of luminance, contrast, and structure. It computes a local spatial index in the terms of average pixel values $\bar{x},\bar{y}$; standard deviation $\sigma_{x},\sigma_{y};$ and covariance $\sigma_{xy}$:

(4) $$PSNR=10log_{10}\frac{{\left(2^{n}-1\right)}^{2}}{\sqrt{MSE}}$$

(5) $$SSIM=\frac{4\sigma_{xy}\bar{x}\bar{y}}{\left(\sigma_{x}^{2}+\sigma_{y}^{2}\right)\left[{\bar{x}}^{2}+{\bar{y}}^{2}\right]}$$

### 3.5. Close-Range Photogrammetry and Structural Health Monitoring

After processing the photogrammetry images through resampling algorithms and assessing their quality using the index metrics, they are analyzed following the procedures explained in Section 2.2. The internal and external parameters in photogrammetry are determined based on the compressed reference and search images captured by the two cameras system. When the compressed images are successfully analyzed and the full-projection matrix is computed, the SHM is performed. SHM images are also compressed following the framework in Figure 3 before processing to generate time signals. The sub-pixel pattern matching method is used to track the location of the optical targets within image sequences. The SHM accuracy is computed based on the difference between the optical target measurement and reference values as the maximum error or RMSE based on the experiments. 

## 4. Compression Framework Implementation and validation using One-Inch Block Experiment

### 4.1. Test Setup

**Figure 4:**
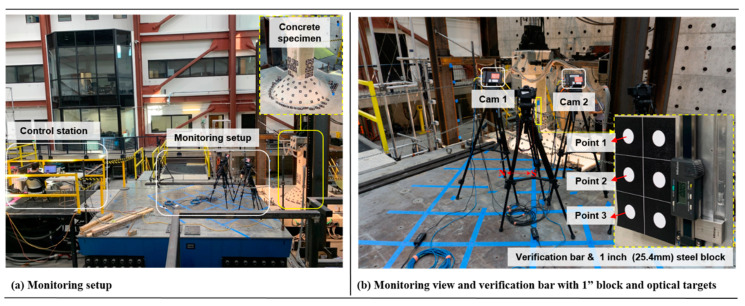
Test setup for validation of the proposed image compression method using a standard 1-in (25.4-mm) block inserted into a sliding verification bar.

The proposed image compression framework was first experimentally evaluated through a one-inch steel block test in the Earthquake Engineering Laboratory (EEL) at the University of Nevada, Reno (UNR). The test considered a sliding bar that can be displaced, along with selected number of targets, when a standard NIST-certified one-inch block is inserted as illustrated in Figure 4. It is noted that the sliding bar was installed onto a reinforced concrete (RC) column and slab subassembly that was being monitored as part of another study. For the best and widest field of view, the vision-based monitoring was set on a higher platform, basically the top of one of the shake tables in EEL, approximately at 5 m from the specimen as shown in Figure 4. The system used two monochrome cameras with the specification listed in Table 1. 

Optical targets were printed on adhesive stickers and carefully attached on both the specimen and the sliding validation bar to avoid losing the targets during test. The radius of the white targets was 21 mm. With camera setup of approximately 1 m separations, all distributed optical targets on the specimen were successfully captured by the cameras. These targets were illuminated by two additional lights. A total of 76 photogrammetry images, 38 captured by each camera, was taken with different position and orientation towards the specimen following the procedure described in Section 2.2. 

The validation test model has the following components: A sliding validation/verification bar as shown in Figure 4 was attached to the RC specimen. For simplicity, the validation test was conducted only on the bar that was displaced using the standard block. The distributed targets on the concrete specimen were used for another set of static experiment but the photogrammetry process was conducted using all targets on the specimen to satisfy the requirements of minimum targets in each photogrammetry images for bundle adjustment convergence. Six optical targets, that were attached to a plate glued to the moving part of the sliding bar as shown in Figure 4, were monitored for the validation test. Three static pictures were taken to validate the vision sensor measurement: initial without steel block, with steel block inserted onto the bar, and final after steel block was taken off the bar. When the steel block was attached, the aluminum plate with the glued optical targets was displaced precisely at 1 inch (25.4 mm) following the dimension of the steel block. The pictures taken with and without inserting the steel block were used as the reference initial and end-state of the validation test. They measured the value of zero as no movement was induced on the optical targets when the static pictures were taken. Therefore, the accuracy measured from this test was based on an absolute single value of 25.4 mm in which the value was compared with the three optical targets measurement shown in Figure 4. 

### 4.2. Compressed Image Quality

#### 4.2.1. Histogram and Output Image Visualization

**Figure 5:**
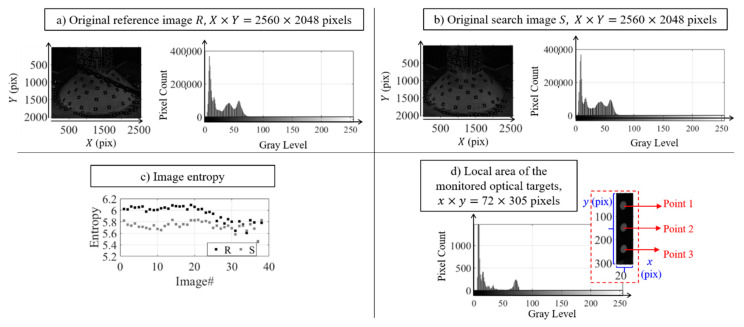
Sample histogram from original-size (full-resolution) images from both cameras: (**a**) reference image R, and (**b**) search image *S*; (**c**) entropy of 76 photogrammetry images; and (**d**) local area x×y= 72 × 305 pixels of three optical targets, all from the one-inch block experiment.

Before analyzing the photogrammetry results, the compressed photogrammetry image quality was first assessed to check whether the images were not distorted nor blurred due to the applied resampling algorithms. It was also important to observe whether the input image information was still preserved in the compressed image. The example of the photogrammetry image with original size of 2560 × 2048 pixels is shown in Figure 5. 

The histogram shows that most pixels have rather low intensity values that the pixel values are not distributed over the entire gray level intensity range. From the histogram, the inferred photogrammetry image information indicates that the captured image was a low-contrast image, i.e., the object being viewed was mostly dark in color and located on a dark environment. Also, the specimen was illuminated with low-intensity light. More details are observed when the image is zoomed into a local area of 72 × 305 pixels surrounding the three optical targets or points used for accuracy assessment purpose as shown in Figure 5. The figure shows that the low-pixel intensities are not only a result of the dark environment but the black color of the optical targets as well. The vision-based sensor setup was designed such that most of the image information was used as the background for the ease of separation with the white dots of the optical target. Even though the brightness level of the white dots were low, for example it was within the range of 60–80 intensity level as shown in Figure 5, it was still able to be identified in the photogrammetry process.

**Figure 6:**
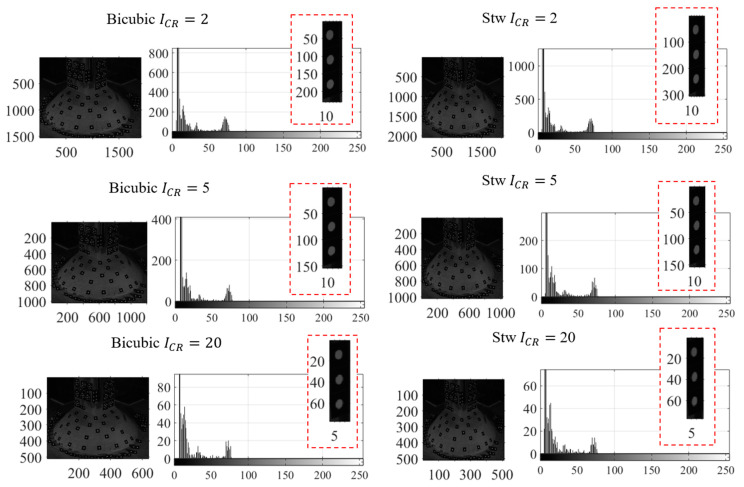
Compressed images from non-adaptive linear interpolation using Bicubic algorithm and wavelet transform using Stw compression for three compression ratios ($I_{CR}=$ 2, 5, and 20) with histograms of the local area of the monitored optical targets from the 1-inch block experiment.

The photogrammetry images were compressed by the resampling algorithms from the original size of 2560 × 2048 pixels to 75% (1920 × 1536 pixels), 50% (1280 × 1024 pixels), and 25% (640 × 512 pixels) of its original size. These percentages were selected to keep the compressed images size as integers. The data size after compression were reduced from 5.2 MB for a single image to 2.6 MB, 1.04 MB, and 250 KB for 75%, 50%, and 25% resampling, respectively. These data size reductions were associated with compression ratios $I_{CR}$ of 2, 5 and 20. The examples of the photogrammetry image after compression using non-linear adaptive interpolation and wavelet transform are shown in Figure 4. The clear observation is that the dynamic pixel counts of the input (original) image is also reduced to a smaller number of pixels as the effect of quantization during the compression. The compressed image also does not contain gray values that did not exist in the input image as indicated by the histogram of the local area surrounding the three points in Figure 6. The gray level intensity of the compressed image using both resampling methods is still within the range of the input image intensity level. 

**Figure 7:**
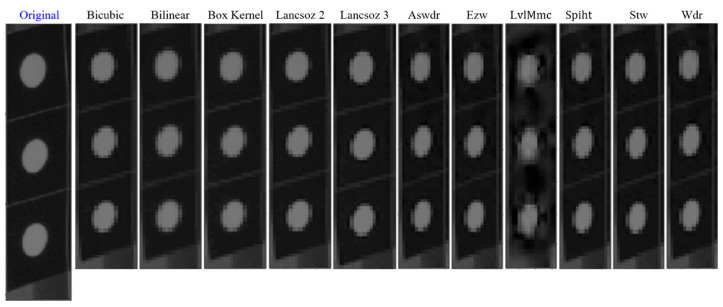
Comparison of a high quality input image (original size) against compressed versions at $I_{CR}=$ 20 obtained using different non-adaptive linear interpolation and wavelet compression (images enlarged by 400% with +20% and −40% adjustment of brightness and contrast for better visualization).

By inspecting the histogram of the local area of the three optical targets with $I_{CR}=$ 2 in Figure 6 both resampling methods resulted in identical histogram of their compressed image. Besides the quantization effect on the number of pixels per gray level intensity, no spatial artifact occurs in low compression ratio images, but it comes into effect in higher compression ratio $I_{CR}=$ 20 as shown in Figure 7. The output of the resampling creates the pixels surrounding the edge to look averaged, so it appears as a large block, which is generally known as staircase noise in photography. By enlarging the image size, this effect on the sharp edges becomes visible especially on the edge of the white dot. Another spatial artifact shown in Figure 7 is the color bleeding where the edge of the white dot overlaps or bleeds into another gray level intensity. This artifact appears in all compressed images using either non-adaptive interpolation or wavelet compression, but the most apparent color bleeding was shown when using the coefficient threshold (LvlMmc) for wavelet transform. Therefore, the color bleeding intensifies the pixel counts in several gray level as shown in their histogram in Figure 6. However, the intensity still falls within the range of the input image, i.e., the gray level information from the input image is still preserved. When the brightness and contrasts of the image are enhanced as shown in Figure 7, small artifacts around the edge of the white dots are visible, shown as dots or so-called ringing or edge halos. They were formed due to the loss of sharpness as the output image from the compression oscillated at a fading rate around the sharp edges during the intensity transitions.

Overall, the image compression from the resampling methods using either non-adaptive linear interpolation or wavelet compression experiences reduction of pixel counts within the distributed gray level intensity. The output images also show spatial artifacts such as staircase noise, color bleeding, and edge halos. These artifacts are left untreated, as this study is not intended to enhance or to correct any additional features that are visible after image compression. The photogrammetry as well as SHM procedures were continued using these compressed images to understand the implications of the image compression as discussed in Section 4.3 and Section 4.4.

#### 4.2.2. Quantitative Analysis Results

**Figure 8:**
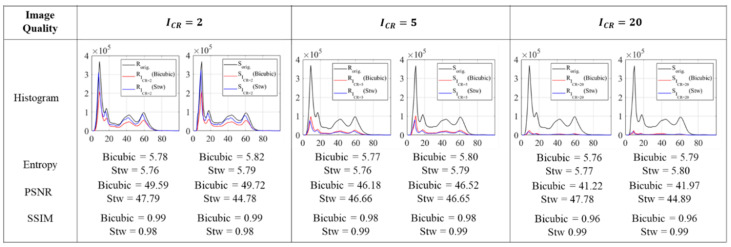
Compressed images histograms from reference, R, and search, S images using bicubic interpolation and spatial orientation tree wavelet (STW) resampling methods for $I_{CR}$ = 2, 5, and 20 and quantity index metric (the gray intensity value range is selected between 0 to 100 for better visualization).

Quantitative analysis was conducted on all compressed photogrammetry images with the total of 2508 images (76 images × 3 compression ratios × 11 resampling operations). Two examples are shown in Figure 8 for using the resampling output of bicubic interpolation and spatial orientation tree wavelet. The histogram focuses only on the predominant gray values between 0 and 100 intensity captured by the two cameras. The figure shows the similarity of gray intensity value distribution between original and compressed images without any intensity outside the input image range. The pixels count was also identical between reference and search images for each compression rate. 

Image entropy defines the randomness contained in the image, so it directly relates to the image histogram. The maximum entropy is equal to the number of bits n, i.e., 8. It can be achieved only if the histogram uses all the available dynamic range from 0 to 255 with equal probability of the pixel counts, or to visualize, the resultant probability distribution is completely flat. The entropy of all the 76 images used in photogrammetry with original size is shown above in Figure 5c. The input images that are associated with the example in Figure 8 have entropies of 5.78 and 5.82. After compression, their entropies do not change significantly, even at higher compression ratio, as they are within the range of 5.76−5.78 and 5.79−5.82 for the reference and search image, respectively. This agrees with the visualization from the histogram in Figure 8 where the applied resampling algorithms only creates quantization without changing the gray level distribution or adding additional intensity correspond to the input image. Moreover, a quick example is provided to confirm the observation previously mentioned regarding the lower and upper bound value of compression ratios based on the Shannon entropy. Using the relationship mentioned in Section 3.4, with the entropy of the original size image of 5.78 for example, the lower and upper bound compression is computed as $\frac{8}{5.78+1}<I_{CR}<\frac{8}{5.78}$. Therefore, such image can be compressed within the range 1.18$\;<I_{CR}<$ 1.38, which provides very low values for compression that will not be of benefit. However, the compression ratios are still stretched to 20 in this study, and hence, recommend not to use such low theoretical values.

**Figure 9:**
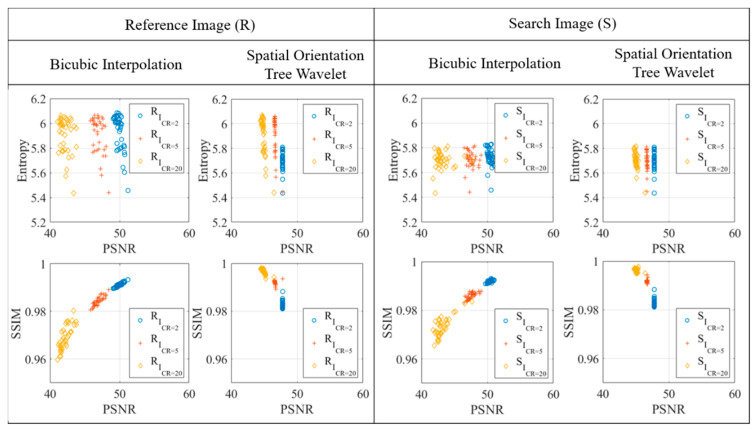
Evaluation of bicubic and STW compression quality of 76 images (38 each for reference and search images) used in the photogrammetry step.

The peak-signal-to-noise ratio PSNR and structural similarity index metric SSIM of the sample compressed images from the bicubic and STW wavelet are also shown in Figure 8. Moreover, the complete results of the compressed image entropy, PSNR, and SSIM used in the photogrammetry from those cases are shown in Figure 9. PSNR measures the quality of the reconstructed image where the noise is the error introduced by the resampling algorithm. For all photogrammetry images compressed using bicubic and spatial orientation tree wavelet, the results show a high PSNR more than 40 dB, as shown in Figure 9. As for SSIM, a good quality index is implied in Figure 9 where the SSIM for all photogrammetry images is more than 0.96. The assessments based on entropy, PSNR and SSIM show that the compressed images still have a good reconstruction quality with insignificant noise even at a higher compression ratio.

The relationship between PSNR with entropy and SSIM based on the applied compression ratio is shown in Figure 9 using bicubic and STW compressed images. It is observed that the compression rate slightly reduces the values of PSNR either by using bicubic interpolation or STW compression. The PSNR distribution using STW wavelet is more concentrated into a constant value while bicubic interpolation generates more variations in the PSNR distribution.

The relationship between image entropy and PSNR as shown in Figure 9 is somewhat constant that the change of entropy does not significantly affect the change of PSNR. A non-linear relationship is observed between PSNR and SSIM that is more visible when using bicubic interpolation. An upward sloping is shown on the bicubic interpolation curve suggests that increasing the PSNR also increases the SSIM value. Meanwhile, the PSNR and SSIM relationship trend when using STW compression is not as noticeable as with bicubic interpolation, only that the SSIM improves for higher compression ratio, whereas the PSNR declines once the compression ratio is higher. 

### 4.3. Effect of Image Compression on the Photogrammetry Process

The compressed images from non-adaptive linear interpolation and wavelet compression were processed following the procedure of the close-range photogrammetry outlined before in Figure 3. Using $I_{CR}=$ 2, 5, and 20, the photogrammetry was successfully conducted as both internal and external orientation parameters were possible to be identified from the compressed images. This shows that even though the output of the image compression has some artifacts (e.g., more visible defects from the LvlMmc method at $I_{CR}=$ 20), the photogrammetry still yields meaningful and acceptable results. In this section, the photogrammetry results such as principal point $u_{0},v_{0}$, radial symmetric, asymmetric and tangential distortions *A*_1_, *A*_2_, *A*_3_, $B_{1},B_{2}$ from compressed images are presented. In addition, the effect of image compression using a selected resampling method is also explored in the context of relating the change of photogrammetry parameters with respect to the applied compression ratio. Also, for a given compression ratio, photogrammetry results using the compressed images are checked to see whether there is any trend (e.g., specific ratio or scale) when compared to results from the original full-resolution input images.

#### 4.3.1. Principal Point Location in the Compressed Image Plane

**Figure 10:**
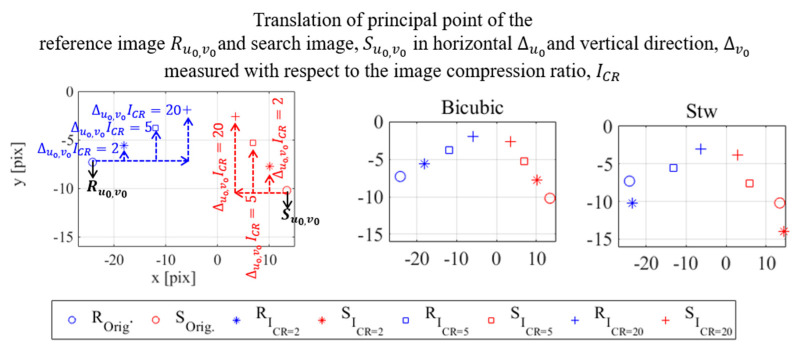
Principal points $u_{0},v_{0}$ location in the input image plane and their translation in the compressed image plane for different compression methods and ratios. The examples are shown for the bicubic interpolation and STW compression methods.

The photogrammetry results of the one-inch block test using input images with the size of 2560 × 2048 pixel are given in Table 2. The table shows that the locations of the principal point in the x and y direction $\left(u_{0},v_{0}\right)$ are −24.17, −7.30 and 13.44, −10.21, for the reference R and search image S, respectively. These positions are plotted in Figure 10 followed by the principal point results using compressed reference R and search S images from different compression methods and for the three different compression ratios. For convenience, the figure shows only pixel range where the principal points are located, instead of the entire image resolution. In all locations, the units of the principal point in both axes are in pixels. Figure 10 clearly shows that the principal point is shifted in the horizontal and vertical axes along with the applied compression ratio. Although the photogrammetry procedure is similar for all compression ratios, different compression algorithms seem to affect the location of the principal point. This shifting is expressed by a pixel translation ratio in both axes, $\Delta_{u_{0}}$ and $\Delta_{v_{0}}$, which is the ratio of a principal point coordinate in the original image with respect to its compressed version (see Figure 10 for an illustrative example). The computed translation ratios are summarized in Table 3. The table shows a varied behavior of the principal point translation between non-adaptive linear interpolation and wavelet compression methods. The principal point translation ratio in the image plane with $I_{CR}=$ 2 ranged between 0.66−1.34, which increased for $I_{CR}=$ 5 to 1.16−2.51. Translation ratios become even larger for $I_{CR}=$ 20 where a principal point coordinate is shifted significantly within a translation ratio of 8 when the Aswdr compression method is used for instance. It is also noted that the principal point is shifted more in in the horizontal x direction compared to the vertical y direction, even though the image is resampled using a similar scale in both directions.

#### 4.3.2. Optical Target Locations in the Compressed Image Plane

A total of 88 optical targets distributed on the RC specimen and verification bar (see Figure 4) were used in the photogrammetry procedure. Therefore, the output listed in Table 2 was in fact the result from the bundle adjustment of all these targets on the 76-photogrammetry images. It is also noted that all 88 targets were available to be used for SHM purpose. However, the one-inch block test used in quantifying the measurement accuracy was conducted using only three optical targets placed on the sliding bar as mentioned before. Accordingly, only these three targets or points were used to assess the effect of applied compression on their 2D position on the image plane. 

**Figure 11:**
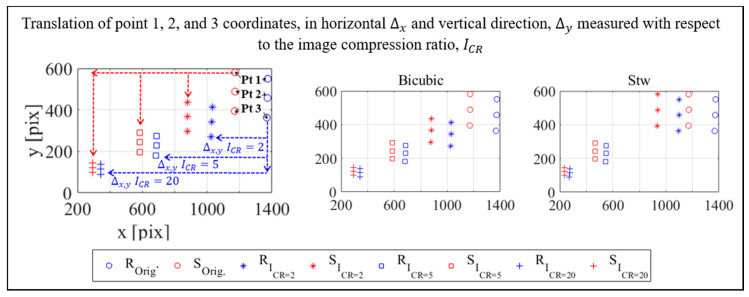
Points 1, 2, and 3 location in the input image plane *x*,y and their translation in the compressed image plane,$\;\Delta_{x,y}$ for different compression methods and ratios. The examples are shown for the bicubic interpolation and STW compression methods.

One of the outputs of the photogrammetry is the 2D location of the optical targets, i.e., the horizontal x and vertical y pixel coordinates in the image plane. The results when the compressed images are used is shown in Figure 11 and they are plotted together with the 2D plots of the three points when the original size images are used in the photogrammetry. A much clear clustering of these points is observed and a definite 2D translation of their positions from the original location (uncompressed) is presented. The change of target position in the original and compressed image is again expressed by the translation ratio as shown in Table 4. However, this time the ratios are shown to be the reciprocal of the applied scaling of the digital image when non-adaptive linear interpolation is used, i.e., 75% compression leads to a translation ratio of 1.30, and 50% or 25% leads to 2.0 and 4.0, respectively. The results from wavelet compression are different only because the horizontal axis of the input image needs scaling to fulfill the requirement that the input image size must be the powers of two. Based on the results shown in Table 4, it can be confirmed that the 2D point translation in the image plane based on the compressed image photogrammetry using both resampling methods is in fact the inverse of the scaling factor applied on the input images.

### 4.4. Effect of Image Compression on the Vision System Measurement Accuracy

The objective of the previous subsections was to confirm that the conducted image compression could still lead to a successful photogrammetry process, i.e., internal camera parameters could be determined for instance. This was still true regardless of some variations between the different compression methods, that have been quantified (e.g., principal point or optical target translation). However, such previous results do not necessarily represent measurement accuracy after image compression, which in turn, is the focus of this subsection. Using same compression methods as before, the input images for the 1-inch displaced targets were compressed then used to determine the displacement value. The value used for validation here was the absolute 1 inch (25.40 mm) resulting from inserting the one-inch block onto the sliding bar. Using original image size subpixel registration, the displacement of point 1, 2, and 3 as shown in Figure 4 was measured as 25.48, 25.46 and 25.48 mm, respectively. The measurement accuracy was assessed by computing the average error of these values with respect to the absolute value of 25.4 mm. The error was computed as 0.31%, 0.24%, and 0.31% for points 1, 2, and 3, respectively, and the average error from all three point was computed as 0.28% when uncompressed images were used.

The effect of image compression on the vision-based displacement measurement is expressed using two metrics. These are the error of the three points with respect to two reference values: (1) the measurement using the original size image, i.e., 25.48, 25.46 and 25.48 mm for points 1, 2 and 3, and (2) the absolute value of 25.40 mm. Table 5 provides all the obtained measurement values from different compression methods. In addition, the measurement result with respect to the input original image computed as the average error of the three points, $\Delta_{avg}$(%), is summarized in Table 5.

The results show a very high accuracy at lower compression ratio, especially when the images are compressed using non-adaptive linear interpolation method. At higher compression ratios, the accuracy becomes slightly less and the measurement using compressed images using wavelet shows relatively higher error as compared to the original values. Similar trends are observed when comparing the measurement from compressed images with the absolute value of 25.4 mm. The accuracy measured at lower compression ratio is very close to the accuracy given by the input image, i.e., 0.28% error. The maximum error is shown to be associated with the images compressed using LvlMmc method as 0.96% error with respect to the absolute value of 25.4 mm. Recall the example of compressed image with $I_{CR}=$ 20 using Lvlmmc method in Figure 7; that relatively lowest quality output image possibly contributed to the lower measurement accuracy using this method. Thus, future studies can further examine and relate the gray value distributions on the output images from high compression ratios to displacement computation using sub-pixel image registration algorithm.

Overall, the results shown in Table 5 provide the ultimate validation and verification for adopting image compression using either linear interpolation or wavelet transform methods, where displacement measurement errors can be less than 1%. There are no clear trends on which algorithms under the two methods would be preferred for higher accuracy. However, the non-adaptive linear interpolation methods could be argued to provide slightly more accurate results than the wavelet transform methods. Nonetheless, the table highlights the specific cases that can be recommended as best algorithms based on error values. Given these promising results obtained from the simple 1-in block test, it was desired to extend the study to more realistic cases including full-scale building vibration monitoring, which is the focus of the next section.

## 5. Accuracy Assessment of Image Compression Framework Using Static and Dynamic Tests

The implications of image compression in the photogrammetry process and the vision-based sensor metric accuracy was evaluated in Section 4 using a precisely known single-value test. i.e., the one-inch block test. In this section, the accuracy of vision-based sensor metric using compressed images is further evaluated. Results from two recently conducted laboratory tests were used. The first case study applies image compression on quasi-static test images. The second case study implements the proposed framework on monitoring images from a signature full-scale three-story RC building tested under seismic loads at the world’s largest shake table, located in Japan. While different compression methods and algorithms were used and compared in the previous section, only the Bicubic interpolation was selected to use for the two case studies discussed in this section due to its low-computational efficiency and better accuracy as evaluated in Section 4.

### 5.1. Quasi-Static Experiment

#### 5.1.1. Experimental Setup and System Configuration

**Figure 12:**
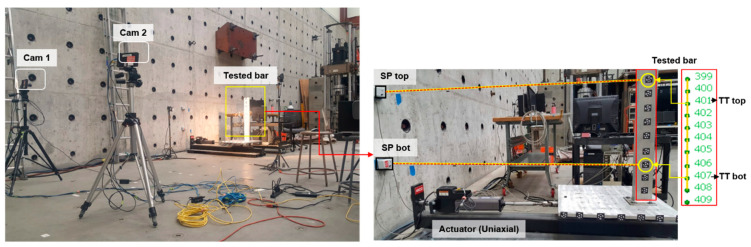
Quasi-static test setup used for experimental validation of image compression along with the vision-based sensor components and optical targets details.

The accuracy of image compression for displacement measurement was experimentally evaluated through a more elaborate validation quasi-static test. A small ANCO shake table was used to conduct uniaxial loading for a rigid test model at the Earthquake Engineering Laboratory at the University of Nevada, Reno. The system configuration of the vision-based sensor system using two cameras is same as used in the one-inch block test (Table 1). For this test, the sampling rate was 32 fps and monitoring/recording of the test continued for 33.5 s, which resulted in a total of 27.4 GB data size. The test model was an aluminum block specifically constructed for this study with the dimensions 100 mm × 10 mm × 900 mm as shown in Figure 12. In the experiment, the shake table was moved manually by pushing the specimen while setting the actuator free to follow the motion. The test setup is shown in Figure 12 along with the distribution of the optical targets. For the purpose of photogrammetry, 11 optical targets (numbered from 399 to 409) were attached to the specimen and six targets (numbered 410 to 416) were distributed on the shake table. The targets were printed from photogrammetry software [99] on adhesive stickers and carefully attached to avoid losing the targets during the tests. The radius of the white circular targets was 3.5 mm. For the best and widest field of view for the test, the monitoring station was located approximately 3.8 m from the specimen. Using a camera setup as shown in Figure 12 with approximately 1.77 m of camera separations, all distributed targets were successfully captured by the cameras. A total of 64 images (32 on each camera) were used in photogrammetry and the results using the original and compressed image size are given in Table 6. With the given vision sensor configuration and number of images, the photogrammetry could be still processed for high compression ratios. The static accuracy assessment in this experiment is provided only for the maximum applied compression ratio, i.e., $I_{CR}=20$.

#### 5.1.2. Static Accuracy Assessment of Vision-Based Sensor Metric

For the validation quasi-static test, monitoring was conducted with only a 32 fps, i.e., 32 Hz sampling rate, and global electronic shutter of 3000 msec. The test utilized the full resolution ROI of the cameras, i.e., 2560 × 2048 pixels. Total images recorded from the test were 2378 images associated with the data size of 27.4 GB. For the validation purposes, two NIST-calibrated string potentiometers, namely SP top (top channel) and SP bot (bottom channel) were connected to the specimen as shown in Figure 12.

**Figure 13:**
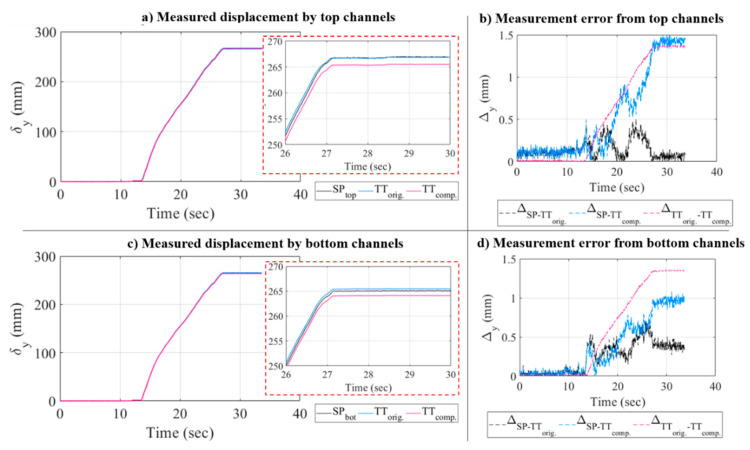
Comparison of measured displacement from SP and target-tracking (TT) using original and compressed images and the relative error between different measurements from quasi-static test.

SP top was connected to the specimen close to optical target #401 (TT top) and SP bot was connected close to target #407 (TT bot) for comparison purposes. The displacement measurements from both optical targets and string potentiometers were compared, and the error was computed as the percentage difference between SP and optical target measurement. With $I_{CR}=20$, the test data size was possible to be compressed to 1.37 GB. The processed displacement history of the static test as obtained from both original and compressed images with $I_{CR}=20$ are shown in Figure 13, which are also compared to the SP measurements.

**Figure 14:**
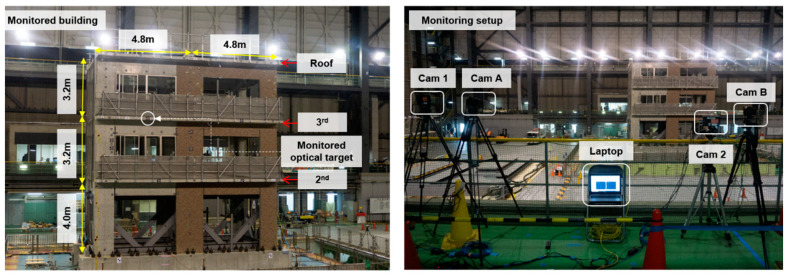
E-Defense full-scale RC building test and monitoring setup using two vision-based sensor systems. The optical target enclosed in white circle is used in this study to obtain seismic displacement and relative measurement accuracy using original and compressed images from both sensor systems.

The measurement errors of the results shown in Figure 14 were calculated and listed in Table 7 for the top and bottom channels. Three accuracy or error metrics are determined based on the selection of reference sensor. Using string potentiometer for reference, the accuracy of target-tracking (TT) from original and compressed images is estimated with respect to the SP values. Another error is estimated using original image results as reference for the compressed image results. Using the original images, the vision sensor metric is shown to have 0.51% and 0.27% error with respect to SP at the top and bottom, respectively. The root mean square error (RMSE) associated with these errors, when calculated in actual measurement units (mm), is 0.18 mm and 0.31 mm for the top and bottom channel measurement, respectively. Using compressed images, the RMSE slightly increased to 0.75 mm and 0.52 mm for respective channels. These results show that from the continuous static monitoring, using compressed images only slightly reduce the accuracy of the static measurement. When comparing the results between vision sensors metric using original and compressed data, RMSE of 0.79 mm is measured at both monitoring locations. The maximum relative error between using compressed and original images is shown to be only around 0.5%. Therefore, similar to the results from the one-inch block test, the accuracy of using compressed image for static displacement measurement is very acceptable. For comparison purposes, the maximum error is found to be about 0.3% and 0.5% when bicubic interpolation is used for image compression for the one-inch block and quasi-static continuous test images, respectively.

### 5.2. Seismic Shake Table Experiment

#### 5.2.1. Vision-Based Sensor System and Monitoring Setup

The last part of this study is concerned with assessing the accuracy of image compression in measuring dynamic vibration and displacements of actual structures. For this purpose, a signature seismic shake table test of a full-scale three-story RC building shown in Figure 14 is used. The test was performed in December 2019 at the world’s largest shake table facility, E-Defense, located at the National Research Institute for Earth Science and Disaster Resilience (NIED) in Kobe, Japan. This test was part of the Tokyo Metropolitan Resilience Project Subproject C [67] and the authors were involved through a US-Japan collaboration to monitor the test using vision-based systems. The general purpose of the test was to improve the resiliency of buildings and to develop SHM techniques that could rapidly assess the safety and post-disaster functionality of buildings after major earthquake events. More details about the project and the building system can be found in Yeow et al. [100].

To monitor the building vibration and seismic performance, SHM was conducted using two-types of vision-based sensor systems. The first system used the same high-speed cameras as in the one-inch block and quasi-static tests, which is shown as Cam 1 and Cam 2 in Figure 14. The second system used consumer-grade digital cameras that was set to monochrome mode, which is shown as Cam A and Cam B in Figure 14. No additional lights were used in the monitoring, so both vision systems relied completely on the ambient light sources and the setting adjustment in each camera. Thus, it is noted that the captured photogrammetry images as well as the test images required image processing to enhance their low-level of brightness and contrast before resampling. Since image enhancement is not the scope of this study, the process is not provided here, and only discussion on the image compression effects on dynamic measurements of vision systems is provided here. Table 8 provides more details on the two vision sensor systems and their configuration. Both systems used CMOS sensor. However, the .jpg format was used as opposed to the .tiff for the digital cameras versus the high-speed cameras and the input image resolution was unchanged. A lower resolution was set in the digital camera even though it could shoot image at the maximum of 5184 × 3456 pixels (18 MP). The reason was that the video recording mode was used for the seismic test monitoring instead of continuous burst image recording. Therefore, image resolution of 1920 × 1080 pixels was set for photogrammetry process to be similar with the 1920 × 1080 full HD video. The sampling rate for the seismic test was selected as 32 fps for the high-speed cameras, which resulted in 7680 images for 120 sec recording duration and the total data storage of 38.4 GB in both cameras. The digital camera monitored the tests through video recording with the HD quality of 1920 × 1080 pixels. After video processing, the total image data storage for the system was computed as 2.3 GB.

A total of 50 photogrammetry images was captured by each vision sensor system using their respective input image resolution listed in Table 8. Since the image compression ratio of 20 could be achieved in the previous experiments, similar step was also attempted using the photogrammetry images for the seismic tests purpose. However, the optimum compression ratio that could be achieved by images from both vision systems was found to be $I_{CR}=5$. When compression ratio of 20 was applied and used in the photogrammetry, the bundle adjustment process failed and could not be completed. In short, the reason for the failed bundle adjustment in the photogrammetry process could be attributed to relaxing the target identification threshold to 2.0 pixels, which led to random recognition of some natural features such as window reflections but only in some frames. Selected results of the photogrammetry, i.e., principal point location, from both monitoring systems original and compressed images are listed in Table 9. It is noted that image compression output using $I_{CR}=2$ and $I_{CR}=5$ for the digital vision system is 1440 × 810 pixels and 960 × 540 pixels, respectively. As for the high-speed cameras, same output image size as previous tests was used.

**Figure 15:**
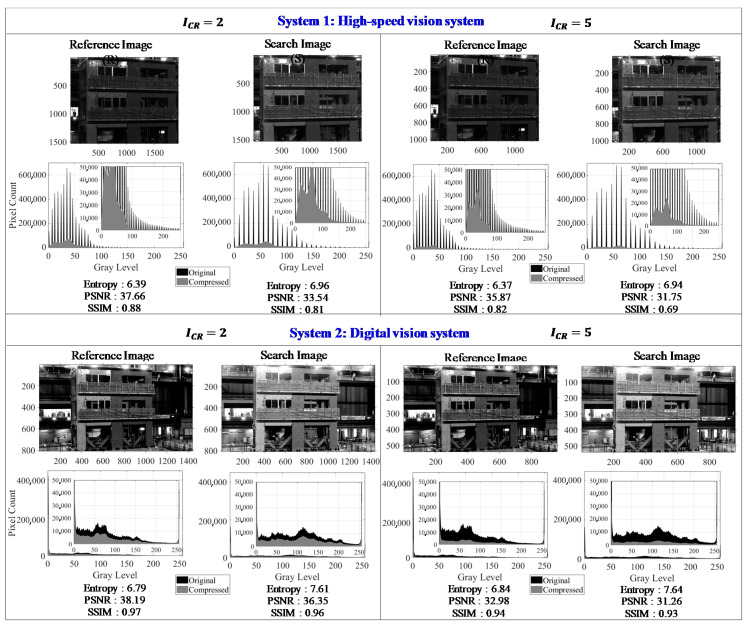
Image quality assessment from compressed images using bicubic interpolation measured by two-vision-based systems, i.e., high-speed vision system and digital cameras system.

An example of the compressed image histogram and the quality index metric for both sensor systems is shown in Figure 15.

The histogram of the high-speed vision system clearly shows the stretching as the effect of image enhancement. The reduction of the pixel counts is also observed once the compression is applied to the image. The image entropy measures higher values due to the applied image enhancement process and values lower than 40 dB are computed for the PSNR. The SSIM results infer that the structural information of compressed images slightly matches that of input images. A larger difference is observed at the higher compression ratio where the computed SSIM is 0.69 using the high-speed vision systems.

From Figure 15, it is shown that a better similarity index is measured from the digital cameras system as the SSIM is computed to be more than 0.90. As for the entropy and PSNR results, the values are closer to the results from the high-speed vision system. One noticeable visualization from the histogram of compressed image from digital vision system is the high peaks at the beginning and end of the gray level bins as shown in a zoomed-in view in Figure 15. The image enhancement causes some region in the image as pure black and white, or also called clipping. The gray values of this specific region are outside the sensor dynamic range so they are set as the minimum (0) and maximum (255) and appear as the peaks in the histogram bins. These peaks provide no detail related to the image information, so the region associated with these peaks will not expose any features even if there is an optical target attach in the region.

#### 5.2.2. Dynamic Accuracy Assessment of Vision Sensor Metric

**Figure 16:**
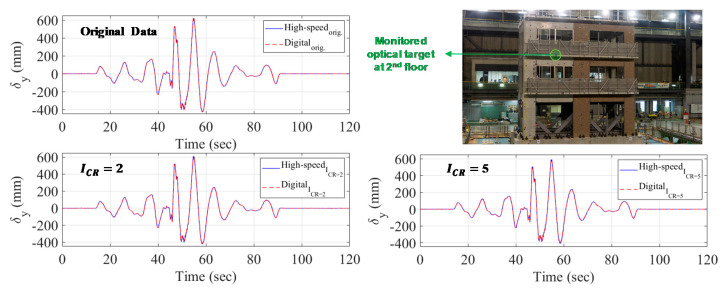
Displacement history from low-intensity seismic test as obtained from original and compressed images from two different vision-based systems (high-speed and digital cameras).

Low-to-high amplitude of seismic waves, also known as ground motion excitations, were applied to the RC building. White noise excitations were also applied in between the seismic tests. A sample of the processed results, i.e., displacement history at the target identified in Figure 16, from TT under a low-amplitude (20% scale of a Japanese synthetic ground motion [65]) seismic excitation is shown in Figure 16. The high-speed camera is chosen as the reference sensor and three types of dynamic assessment metrics for the vision systems are evaluated as explained next.

For error assessment notation, the displacement values measured at the selected optical target (Figure 16) using high-speed cameras with full resolution image and digital cameras with full HD resolution are denoted as $a_{1}$ and $b_{1}$, respectively. Using compressed images, the displacement values measured by the high-speed and digital systems are defined as $a_{i}$ and $b_{i}$, respectively, and *i* = 2 or 5 for $I_{CR}=2\;\mathrm{or}\;5$, respectively. The first assessment is the “relative error” or $\Delta_{rel,CR=i}$ that is used to define the accuracy of compressed digital camera image results with respect to high-speed camera results (see Equation (6) for maximum relative error calculation). The second assessment is the *“absolute error”* or $\Delta_{abs,CR=i}$ that is used to define the accuracy of original and compressed image results from the digital cameras system with respect to the reference high-speed system with full resolution images. The maximum absolute error is computed based on Equation (7), and the case when *i* = 1 refers to error in digital camera results relative to high-speed cameras at full-resolution. The last assessment is evaluating the effect of applied compression on each vision system relative to their measurement from full resolution images. The maximum “compression error” is calculated based on Equations (8) and (9) for the high-speed and digital camera systems, respectively:

(6) $$\Delta_{rel,CR=i}\left(mm\right)=\max\left[abs\left(b_{i}-a_{i}\right)\right];\;\Delta_{rel,CR=i}\left(\%\right)=\left(\frac{\Delta_{rel,CR=i}}{a_{i}}\right)\times 100\;;\;i=2,\;5$$

(7) $$\Delta_{abs,\;\;CR=i}\left(mm\right)=\max[abs\left(b_{i}-a_{1}\right)];\;\Delta_{abs,CR=i}\left(\%\right)=\left(\frac{\Delta_{abs,CR=i}}{a_{1}}\right)\times 100\;;\;i=1,\;2,\;5$$

(8) $$\Delta_{comp,a,CR=i}\left(mm\right)=\max[abs\left(a_{i}-a_{1}\right)];\;\Delta_{comp,a,CR=i}\left(\%\right)=\left(\frac{\Delta_{comp,a,CR=i}}{a_{1}}\right)\times 100\;;\;i=2,\;5$$

(9) $$\Delta_{comp,b,CR=i}\left(mm\right)=\max[abs\left(b_{i}-b_{1}\right)];\;\Delta_{comp,b,CR=i}\left(\%\right)=\left(\frac{\Delta_{comp,b,CR=i}}{b_{1}}\right)\times 100\;;\;i=2,\;5$$

Table 10 provides a summary of the peak displacement value as measured from original and compressed images from both monitoring systems. The peak observed displacement measurement from the original full-resolution high-speed and digital systems was 618.88 mm and 615.37 mm, respectively. Using compressed image data, the peak displacement was slightly lower as compared and reported in Table 10. For proper comparative assessment, the different errors defined above as per Equation (6) through Equation (9) are calculated and summarized in Table 11. First, before exploring the image compression effects, the accuracy of both vision systems is assessed using Equation (7) (for *i* = 1) where the high-speed vision system is selected as the reference sensor. The absolute maximum error in the digital system measurement relative to the high-speed system is 6.7 mm (1.08%), which shows that both consumer-grade and high-end high-speed sensor systems are comparable.

The relative error (Equation (6)) in the digital system is reported in Table 11 as 12.73 mm (2.09%) and 18.64 mm (3.15%) for image compression ratios $I_{CR}=2$ and $I_{CR}=5$, respectively. The absolute error of the digital system is also computed for each compression ratio with respect to the high-speed vision system with original data (full resolution images). As inferred from Table 11, the accuracy of the digital system declines, i.e., higher error is observed, at $I_{CR}=5$ where the maximum absolute error is computed as 45.91 mm (7.42%). This absolute error is approximately two times the relative error that is measured as 18.64 mm (3.15%). The last dynamic measurement accuracy metric for the vision-based sensors is concerned with the effect of image compression relative to original images for each sensor system separately, i.e., assessment of each system before and after image compression as per Equations (12) and (13). The error values are also reported in Table 11. For the high-speed system, the image compression accuracy in measuring dynamic response is computed as 8.51 mm (1.38%) and 27.26 mm (4.41%) for $I_{CR}=2$ and $I_{CR}=5$, respectively. Comparable error values are observed in the digital vision system measurement as 14.62 mm (2.38%) and 26.96 mm (4.38%) for $I_{CR}=2$ and $I_{CR}=5$, respectively.

In summary, the results in Table 11 show that it is possible to measure the dynamic behavior of actual vibrating structures and other large-scale systems using compressed images either from high-end high-speed cameras or consumer-grade digital systems. Additionally, the Bicubic interpolation that is computationally efficient is demonstrated to be feasible and effective in compressing digital image for both vision systems.

## 6. Conclusions

This study aimed at implementing two general methods of image compression namely non-adaptive linear interpolation and wavelet transform with several algorithms used for both. Successful implementation and demonstration of vision-based sensors image compression is desired for feasible storage, data transmission, and processing needed for future real-time or near real-time SHM. The study used several carefully designed experimental tests to quantify the effects of image compression on accuracy of vision-based monitoring and measurements. While SHM could utilize both acceleration and displacement measurements, the study focused only on displacements since it is the hardest to capture with high accuracy using vision sensors. Meanwhile, accurate displacements are guaranteed to lead to correct acceleration values as established in literature. The main conclusions and key findings of this study are as follows:

- Image compression is a technique that can efficiently reduce the storage needs of the data collected from vision-based sensors but it still needs to be adopted more in infrastructure and large-scale SHM applications. The non-linear adaptive interpolation is shown to be more practical in application than wavelet compression as it does not require any specification of image size. Therefore, non-linear adaptive interpolation is suitable for the type of displacement measurements and SHM demonstrated in this study and can be applied directly without any additional process. The wavelet compression is also shown to be adequate, but it requires the image size to be within the power of two, which might require some scaling of original images before the compression algorithm is applied.
- The image compression affects the quality of the output images mainly in the total pixel counts, which is known as the quantization effect. However, this study quantified such effect and its implication for the first time. The compressed image quality was assessed using several metrics and results confirmed very adequate reconstruction quality with low noise. Although output images of some compression methods or algorithms display spatial artifacts, they can still be successfully used for close-range photogrammetry and SHM purposes.
- The image compression affects the photogrammetry results, i.e., process of determining internal and external parameters of camera sets, especially in identifying the spatial locations of the principal points and optical targets in compressed image plane. A clear cluster of 2D translations of optical targets is observed in the compressed image plane. However, these translations were found to be related to the inverse of the applied scaling with no adverse effects on failing the photogrammetry process for high compression ratios up to 20.
- The high accuracy of static or quasi-static/continuous displacement measurement from high-end vision systems compressed images is inferred from absolute errors less than 1% for compression ratio up to 20. The accuracy was shown to slightly drop when assessed in full-scale dynamic response monitoring and realistic SHM. To be specific, the static accuracy assessment form the quasi-static continuous test was measured to be within 0.56% error, while that from the dynamic test measures a maximum error of 4.4% when using compressed versus uncompressed images. These values, that have been exclusively determined for the first time through this study, are considered adequate for SHM purposes.
- Overall, it is concluded that image compression does have adverse effects or implications on the close-range photogrammetry and SHM accuracy. The bicubic non-adaptive linear interpolation is shown to be computationally effective and is recommended for vision-based SHM image compression applications. On the other hand, future users are cautioned that measurement accuracy seems to slightly deteriorate when very large compression ratios (e.g., more than 20) are desired or when consumer-grade DSRL cameras are used, as opposed to high-end high-speed dedicated systems, for close-range photogrammetry or SHM purposes. With due care, successful monitoring using highly compressed images will be still possible for a wide range of original digital image sizes and vision system hardware with proper selection of image compression algorithm.

## Figures and Tables

**Table 1:** Vision-based system configuration used for one-inch block verification tests.

Camera Type	High-Speed
Color Mode	Monochrome
Data size (single image, test images)	5.2 MB, 30 MB
Image Format	*.tiff*
Record duration (sec)	N/A (snapshots)
Resolution, w×h	2560 × 2048
f (mm)	35

**Table 2:** Photogrammetry results using 76 images with original size of 2560 × 2048 pixels.

Internal Parameters	$u_{0},v_{0}\;(\mathrm{pix})$	$A_{1}$	$A_{2}$	$A_{3}$	$B_{1}$	$B_{2}$
R	−24.17, −7.30	−4.6 × 10^−9^	−1.3 × 10^−16^	−7.9 × 10^−23^	−8.3 × 10^−8^	−1.0 × 10^−7^
S	13.44, −10.21	−3.8 × 10^−9^	−7.6 × 10^−16^	5.8 × 10^−23^	−8.6 × 10^−9^	−1.1 × 10^−7^

**Table 3:** Principal point translation ratios (calculated as shown in Figure 10) in horizontal $\Delta_{u_{0}}$ and vertical $\Delta_{v_{0}}$ directions for different image compression methods and compression ratios.

Method	$I_{CR}=2$				$I_{CR}=5$				$I_{CR}=20$			
	R		S		R		S		R		S	
	$\Delta_{u_{0}}$	$\Delta_{v_{0}}$	$\Delta_{u_{0}}$	$\Delta_{v_{0}}$	$\Delta_{u_{0}}$	$\Delta_{v_{0}}$	$\Delta_{u_{0}}$	$\Delta_{v_{0}}$	$\Delta_{u_{0}}$	$\Delta_{v_{0}}$	$\Delta_{u_{0}}$	$\Delta_{v_{0}}$
Bicubic	1.34	1.31	1.33	1.32	2.03	1.93	1.96	1.93	4.12	3.82	3.92	3.93
Bilinear	1.34	1.32	1.33	1.34	2.01	1.85	1.95	1.95	4.06	3.56	4.03	3.69
Box Kernel	1.34	1.30	1.33	1.32	2.02	1.96	1.96	1.94	3.57	2.49	5.81	2.48
Lanczos 2	1.34	1.32	1.33	1.33	2.04	1.32	1.98	1.38	3.59	2.39	5.36	2.57
Lanczos 3	1.33	1.31	1.35	1.32	1.97	1.30	2.11	1.36	4.06	3.90	4.01	3.93
ASWDR	1.00	0.69	0.97	0.71	1.91	1.19	2.27	1.19	3.14	2.13	7.98	2.77
EZW	1.02	0.69	0.95	0.71	1.82	1.16	2.43	1.21	3.23	2.07	6.95	2.77
LvlMmc	0.91	0.67	1.18	0.66	1.88	1.17	2.51	1.24	3.23	2.07	6.95	2.77
SPIHT	0.99	0.69	1.00	0.71	1.82	1.16	2.43	1.21	3.23	2.07	6.95	2.77
STW	1.03	0.71	0.93	0.73	1.84	1.32	2.26	1.35	3.82	2.43	4.79	2.64
WDR	1.00	0.69	0.97	0.71	1.84	1.16	2.33	1.22	3.82	2.43	4.79	2.64

**Table 4:** Translation of points 1, 2, and 3 locations in the compressed image plane in horizontal $\Delta_{x}$ and vertical $\Delta_{y}$ directions for different compression methods and ratios.

	$I_{CR}$ = 2				$I_{CR}$ = 5				$I_{CR}$ = 20			
Method	R		S		R		S		R		S	
	$\Delta_{x}$	$\Delta_{y}$	$\Delta_{x}$	$\Delta_{y}$	$\Delta_{x}$	$\Delta_{y}$	$\Delta_{x}$	$\Delta_{y}$	$\Delta_{x}$	$\Delta_{y}$	$\Delta_{x}$	$\Delta_{y}$
Linear interpolation	1.30	1.30	1.30	1.30	2.00	2.00	2.00	2.00	4.00	4.00	4.00	4.00
Wavelet transforms	1.25	1.00	1.25	1.00	2.50	2.00	2.50	2.00	5.00	4.00	5.00	4.00

**Table 5:** Summary of the absolute displacement values (mm) from compressed images along with the average displacement accuracy, $\Delta_{avg}\left(\%\right),$ with respect to results from uncompressed images and absolute displacement accuracy, $\Delta_{abs}$(%), with respect to the one inch (25.4 mm) block value.

$I_{CR}$	Point	Method *										
		(A)	(B)	(C)	(D)	(E)	(F)	(G)	(H)	(I)	(J)	(K)
2	1	25.49	25.48	25.46	25.53	25.49	25.42	25.43	25.49	25.45	25.47	25.43
	2	25.48	25.46	25.50	25.47	25.47	25.49	25.51	25.44	25.48	25.48	25.51
	3	25.48	25.49	25.51	25.47	25.49	25.52	25.52	25.43	25.53	25.47	25.54
	$\Delta_{avg}$(%)	0.03	**0.02**	0.10	0.10	0.03	0.16	0.17	0.11	0.12	0.05	0.19
	$\Delta_{abs}$(%)	0.32	0.31	0.36	0.35	0.33	0.30	0.33	**0.21**	0.34	0.29	0.37
5	1	25.49	25.54	25.59	25.52	25.58	25.60	25.61	25.60	25.61	25.59	25.61
	2	25.45	25.50	25.55	25.55	25.50	25.41	25.44	25.56	25.44	25.44	25.45
	3	25.47	25.47	25.52	25.54	25.57	25.44	25.48	25.40	25.48	25.45	25.45
	$\Delta_{avg}$(%)	**0.06**	0.15	0.30	0.23	0.29	0.29	0.21	0.40	0.21	0.23	0.23
	$\Delta_{abs}$(%)	**0.28**	0.41	0.60	0.53	0.59	0.32	0.42	0.47	0.42	0.36	0.40
20	1	25.48	25.51	25.51	25.49	25.49	25.52	25.53	25.68	25.53	25.60	25.52
	2	25.38	25.41	25.41	25.38	25.39	25.53	25.56	25.63	25.57	25.38	25.54
	3	25.24	25.28	25.27	25.24	25.25	25.22	25.26	25.62	25.26	25.44	25.21
	$\Delta_{avg}$(%)	0.44	0.38	0.39	0.44	0.41	0.50	0.50	0.66	0.51	**0.33**	0.51
	$\Delta_{abs}$(%)	0.35	**0.30**	0.32	0.35	0.32	0.58	0.57	0.96	0.59	0.35	0.59

* (A) Bicubic; (B) Bilinear; (C) Box Kernel; (D) Lanczos 2; (E) Lanczos 3; (F) Aswdr; (G) Ezw; (H) LvlMmc; (I) Spiht; (J) Stw; (K) Wdr.

**Table 6:** Results of photogrammetry using input (original) image size and compressed images.

Internal Parameters	Input Image		$\mathrm{Compressed Image}\;I_{CR}=20$	
	R	S	R	S
$u_{0},v_{0}$ (pix)	4.15, 14.88	14.01, −8.88	0.54, 8.97	1.36, −4.55
$A_{1}$	−5.1 × 10^−9^	−3.6 × 10^−9^	−2.80 × 10^−8^	−2.10 × 10^−8^
$A_{2}$	−6.6 × 10^−18^	−1.1 × 10^−15^	−1.10 × 10^−14^	−2.00 × 10^−14^
$A_{3}$	−1.2 × 10^−22^	1.7 × 10^−22^	−1.90 × 10^−20^	1.10 × 10^−20^
$B_{1}$	−1.6 × 10^−7^	−7.0 × 10^−9^	−3.60 × 10^−7^	−2.20 × 10^−8^
$B_{2}$	−1.3 × 10^−7^	−1.1 × 10^−7^	−2.70 × 10^−7^	−2.80 × 10^−7^

**Table 7:** Different estimates of displacement measurement error for TT using original and compressed images with respect to string potentiometer.

Error		$SP-TT_{original}$	$SP-TT_{compressed}$	$TT_{original}-TT_{compressed}$
**Top channel**	$\Delta_{max}$ (mm)	0.19	1.51	1.38
	$\Delta_{max}$ (%)	0.51	0.56	0.52
	RMSE (mm)	0.18	0.75	0.79
**Bottom channel**	$\Delta_{max}$ (mm)	0.71	1.08	1.36
	$\Delta_{max}$ (%)	0.27	0.41	0.51
	RMSE (mm)	0.31	0.52	0.79

**Table 8:** Vision-based systems configuration for the seismic shake table test monitoring.

Sensor System	#1	#2
**Camera Type**	High-speed	Digital DSLR
**Color Mode**	Monochrome	Color used in monochrome mode
**Data size**	38.4 GB	2.3 GB
**Format**	*.tiff*	*.jpg*
**Record duration (sec)**	120	120
**Resolution,** w×h **(pixel)**	2560 × 2048	1920 × 1080
**Sampling rates,** fs	32 fps	30 fps

**Table 9:** Selected results of photogrammetry (principal point coordinates) for the two-vision sensor systems using original images and compressed images with applied bicubic interpolation algorithm.

Monitoring System		Input Image		$I_{CR}=2$		$I_{CR}=5$	
		R	S	R	S	R	S
**High-speed cameras**	$u_{0},v_{0}$ (pix)	22.10, −15.35	5.37, −13.03	13.4, −11.12	3.88, −9.91	9.86, −11.84	2.10, −9.04
**DSLR cameras**	$u_{0},v_{0}$ (pix)	64.5, −334.3	17.4, −370.6	39.23, −242.08	12.59, −284.82	28.77, −257.86	6.82, −257.22

**Table 10:** Summary of the peak displacement value as measured from original and compressed images from both monitoring systems.

	Original	$I_{CR}=2$	$I_{CR}=5$
High-speed cameras system	$a_{1}$ = 618.88 mm	$a_{2}$ = 610.37 mm	$a_{5}$ = 591.61 mm
DSRL camera system	$b_{1}$ = 615.37 mm	$b_{2}$ = 600.76 mm	$b_{5}$ = 575.96 mm

**Table 11:** Summary of the maximum values for the different calculated assessment errors.

Assessment	Original Full Resolution		$I_{CR}=2$		$I_{CR}=5$	
**Equation (6)**	**n/a**	**n/a**	$\Delta_{rel,CR=2}$ = 12.73 mm	$\Delta_{rel,CR=2}$ = 2.09%	$\Delta_{rel,CR=5}\;$= 18.64 mm	$\Delta_{rel,CR=5}\;$= 3.15%
**Equation (7)**	$\Delta_{abs,CR=1}$ = 6.70 mm	$\Delta_{abs,CR=1}$ = 1.08 %	$\Delta_{abs,CR=2}\;$= 21.24 mm	$\Delta_{abs,CR=2}\;$= 3.43 %	$\Delta_{abs,CR=5}\;$= 45.91 mm	$\Delta_{abs,CR=5}\;$= 7.42 %
**Equation (8)**	n/a	n/a	$\Delta_{comp,a,CR=2}$ = 8.51 mm	$\Delta_{comp,a,CR=2}$ = 1.38 %	$\Delta_{comp,a,CR=5}$ = 27.26 mm	$\Delta_{comp,a,CR=5}$ = 4.41 %
**Equation (9)**	n/a	n/a	$\Delta_{comp,b,CR=2}$ = 14.62 mm	$\Delta_{comp,b,CR=2}$ = 2.38 %	$\Delta_{comp,b,CR=5}$ = 26.96 mm	$\Delta_{comp,b,CR=5}$ = 4.38 %
